# Extraction of single serve coffee capsules: linking properties of ground coffee to extraction dynamics and cup quality

**DOI:** 10.1038/s41598-020-74138-1

**Published:** 2020-10-13

**Authors:** André Eiermann, Samo Smrke, Loïc-Marco Guélat, Marco Wellinger, Anja Rahn, Chahan Yeretzian

**Affiliations:** 1 UCC Coffee Switzerland AG, Bernstrasse 180, 3052 Zollikofen, Switzerland; 2 Institute of Chemistry and Biological Chemistry, Coffee Excellence Center, Zurich University of Applied Sciences, Einsiedlerstrasse 31, 8820 Wädenswil, Switzerland

**Keywords:** Chemistry, Engineering

## Abstract

The objective of this paper is to elucidate the variables that govern coffee extraction from single serve coffee capsules. The study was conducted on 43 *Nespresso* and *Nespresso*-compatible capsules of the same geometry, from all of which the coffee was extracted on the same machine. This allowed the link between a range of coffee and capsule (input) parameters with coffee brew (output) variables to be studied. It was demonstrated that the most efficient way to increase total dissolved solids in the brew is to use more coffee for extraction, and/or to grind the coffee more finely. However, grinding too finely can lead to excessive flow restriction. The most significant new insight from this study is the importance of the proportion of *fines* (particles smaller than 100 µm) regarding the capsule extraction dynamics. Capsules with a higher share of fines, for similar median particle size of the ground coffee, led to longer extraction times. General rules applicable for capsule coffee product development were established, although fine-tuning of parameters for successful capsule coffee extraction remains specific to production line and type of coffee.

## Introduction

The single serve coffee sector for ground coffee is divided into high pressure extraction from capsules and low-pressure extraction from soft pods. It is the fastest growing segment within the larger category of coffee products. The global coffee pod and capsule market combined is expected to grow from USD 15 billion in 2017 to USD 29 billion by 2025 at a compound annular growth rate (CAGR) of 8.5%. The high-pressure extraction capsules segment alone, which is the focus in this study, currently leads the coffee pod & capsule market and was valued at around USD 9.61 Billion in 2017 (63% of the whole single serve coffee segment). The domestic consumption of single serve coffee is currently expected to be increasing due to the lockdowns imposed during the 2020 Coronavirus pandemic.

This growing consumer interest and economic importance of single serve coffee products has led to growing scientific interest but remains a relatively little studied topic. Single serve coffee has been used to study the extraction process, the composition of brews^1–10^ and in the preservation of freshness of the ground coffee in hermetically sealed packaging^11,12^. The single serve coffee sector encompasses a broad definition of packaging options, including pods and/or capsules, and is used to prepare a wide range of coffees from filter to espresso. Different systems vary with shapes and sizes of capsules, with the most common espresso capsule system being the *Nespresso* system. Recently, a surge of *Nespresso*-compatible products entered the market. The compatibles have the same shape as *Nespresso* capsules, but may differ in the material they are made of as well as in the origin and quality of the coffee itself.

The advantage of studying the extraction of *Nespresso* and *Nespresso*-compatible coffee capsule is that the human factor is essentially eliminated from the brewing process. Furthermore, the brewing unit, and coffee bed geometry are very similar for all capsules. When these parameters are handled manually in preparing coffee (e.g. semi-automatic espresso), this results in a higher variability in extraction and cup quality^13^. Therefore, the wide spectrum of *Nespresso* and *Nespresso*-compatible coffee capsule products available on the market provides an excellent platform to systematically study the impact of specific parameters in high-pressure extraction from capsules, while brewing unit parameters are essentially kept constant.

Parameters that typically vary from capsule to capsule, in *Nespresso* and *Nespresso*-compatible systems, are the coffee cultivar, blend, amount of coffee in a capsule, roast profile, internal capsule pressure and grind size, all of which lead to different aroma profiles in the cup^14^. The objective of this study is to elucidate the impact of (input) coffee and capsule parameters on a range of (output) brew variables. From a practical perspective, this study aims to benefit the single-serve coffee industry by aiding in understanding the role of the capsule product design for the extraction process from high-pressure single-serve coffee capsules.

## Materials and methods

### Coffee capsule samples and analysis

Coffee capsule samples were either *Nespresso* capsules or compatible with the *Nespresso* system and were obtained from local supermarkets. A total of 43 different coffees were analysed. The internal pressure in each capsule was estimated by putting the capsule inside a closed vessel and subjecting it to increasing pressure until a visual change in the shape of the capsule foil bulge was observed. At this point, it was assumed the pressure was the same as the internal capsule pressure. Oxygen content inside the capsules was measured using an Oxybaby 6.0 gas analyser (Witt-Gastechnik GmbH, Witten, Germany).

### Ground coffee analysis

The capsules were opened and thoroughly emptied to measure the weight of the coffee per capsule (mean of 3 capsules). The roast level (colorimetric) was determined using a Colorette 3b meter (Probat, Emmerich, Germany). Particle size analysis was performed using an imaging particle size and shape analyser with a dual camera system Camsizer X2 (Retsch Technology GmbH, Haan, Germany). At least 10 g of coffee was used for each particle size analysis, and three measurements were performed per capsule type. Median particle size (X50) was determined as the volume weighted median particle size, based on the particle area projected on the images. The share of fines (Q_100µm_) was defined as the volume share of particles smaller than 100 µm in size. Three particle shape parameters were determined as volume weighted averages, sphericity (SPHT), symmetry (Symm) and aspect ratio (b/l) values.

### Capsule coffee extraction

The coffees were extracted from the capsules using a Krups Inissia XN1005 capsule machine. The weight of the brews was measured on-line during extraction whereby the aim was to reach 40 g by stopping the extraction manually. The resulting brew weight was 40.1 ± 0.3 g (mean ± s.d), with all brews between 39.6 and 40.7 g. The extractions were timed manually from starting the pump to cessation of the pump operation.

### Brew analysis

The concentration of coffee in the filtered brew (0.45 µm filter) was analysed using a VST LAB Coffee III refractometer (VST inc., Tulsa, US), calibrated using a soluble coffee sample prepared to a known concentration as according to the instrument instruction manual to display total dissolved solids (TDS) in coffee brews. The extraction percentage was calculated based on the mean weight of the coffee in each capsule type, brew weight and refractometric TDS measurement.

The sensory test of coffees was performed by three Q-graders (certified Q Arabica Grader, by the Coffee Quality Institute). The sensory quality attributes that were determined were flavour, acidity, body and balance on a scale from 5 to 10. The quality of each attributes was assessed by tasting three replicates of the brew by each of the Q-grader. The final values were based on a subjective consensus of the tasters on the quality score that they give to each samples, rounded to the nearest 0.25 units. The reference of the attribute range was the quality that could potentially be achieved by an espresso coffee extracted as a semi-automatic espresso, where a score of 6 is average quality, and 10 exceptionally high quality.

### Statistical analysis

All data analysis and statistical analysis was performed using R statistical computing (R Foundation for Statistical Computing, Vienna, Austria) using integrated packages. All measurements of brews and capsules (Table 1) were conducted in 3 replicates per each sample.

**Table 1:** Properties of the capsules analysed, ground coffees (X50—volume median mean particle size; Q_100µm_—share of fine particles; Roast - roast level in Colorette Pt unit), brews (TDS – total dissolved solids; Extr. – extraction percentage), and the sensory scores of brews, sorted by ascending median particle size. Each line represents a capsule coffee product. The values with errors represent an average value and standard deviation (n = 3).

Sample number	Input extraction parameters (capsule properties)						Output extraction variables (brew properties)							
	Grounds		Capsule				Brew				Sensory scores			
	X50 µm	Q_100µm_ %	Weight g	Oxygen % (v/v)	Pressure mbar	Roast Pt	TDS %	Weight g	Time s	Extr. %	Flavour	Acidity	Body	Balance
1	255 ± 2	15.8 ± 0.8	6.07 ± 0.06	0 ± 0	257 ± 3	65	3.42 ± 0.08	40 ± 0.3	25.7 ± 1.2	23 ± 0.4	6.50	6.00	6.25	6.25
2	279 ± 2	13.2 ± 1.3	5.93 ± 0.06	0 ± 0	360 ± 22	70	3.58 ± 0.12	39.8 ± 0.3	30 ± 8.2	24.7 ± 0.9	6.00	6.25	6.25	6.00
3	283 ± 1	13.9 ± 0.7	5.43 ± 0.06	0 ± 0	210 ± 5	65	3.36 ± 0.14	40.1 ± 0.2	23.3 ± 6.5	25.6 ± 1	6.25	6.25	6.25	6.25
4	299 ± 1	10.7 ± 0.6	5.53 ± 0.06	0 ± 0	227 ± 3	70	2.87 ± 0.07	40.4 ± 0.3	14.3 ± 2.3	21.7 ± 0.4	6.25	6.25	6.00	6.25
5	303 ± 2	11.8 ± 0.9	5.37 ± 0.15	0.1 ± 0.1	207 ± 3	69	3.14 ± 0.08	40.3 ± 0.8	15.3 ± 1.5	24.1 ± 0.1	6.00	5.00	6.00	6.00
6	312 ± 1	11 ± 0.4	5.47 ± 0.06	0 ± 0	165 ± 0	76	3.04 ± 0.07	40.1 ± 0.2	20.3 ± 4.7	22.8 ± 0.6	6.25	6.50	6.00	6.50
7	321 ± 2	11.4 ± 0.4	5.47 ± 0.12	0.2 ± 0.2	82 ± 8	84	3.19 ± 0.05	40.1 ± 0.4	27.3 ± 9.3	23.9 ± 0.5	6.00	6.00	6.00	6.00
8	331 ± 22	12.7 ± 0.6	5.47 ± 0.06	0.8 ± 0.2	105 ± 10	84	3.06 ± 0.11	40.1 ± 0.2	26.3 ± 4.9	22.9 ± 0.9	6.00	6.00	6.00	6.00
9	340 ± 2	12.8 ± 0.6	5.87 ± 0.06	0 ± 0.1	92 ± 13	81	3.15 ± 0.15	39.8 ± 0.1	23.7 ± 2.1	21.8 ± 1	6.00	6.00	6.00	5.75
10	340 ± 1	11.8 ± 0.4	5.53 ± 0.06	0 ± 0	333 ± 5	82	3.17 ± 0.06	39.9 ± 0.2	36.7 ± 0.6	23.6 ± 0.5	6.00	6.00	6.00	6.00
11	364 ± 1	6.9 ± 0.4	5.2 ± 0	0 ± 0	0 ± 0	76	2.9 ± 0	40.1 ± 0.3	19.3 ± 2.5	23 ± 0.2	6.00	6.50	6.00	6.25
12	365 ± 3	11 ± 0.3	5.67 ± 0.06	0 ± 0	83 ± 0	79	3.1 ± 0.26	39.9 ± 0.3	40.7 ± 9.1	22.3 ± 2	6.00	6.00	6.00	6.00
13	365 ± 1	11.6 ± 0.6	5.7 ± 0.17	0.5 ± 0.1	295 ± 3	80	3.27 ± 0.12	39.9 ± 0.3	26.7 ± 3.1	23.6 ± 0.7	5.00	6.00	6.25	6.00
14	366 ± 2	9.2 ± 0.5	5.4 ± 0	0 ± 0.1	317 ± 5	70	2.97 ± 0.15	40.5 ± 0.5	12.3 ± 1.2	22.9 ± 1.1	6.25	6.25	6.25	6.25
15	367 ± 1	8.1 ± 0.2	5.03 ± 0.06	0 ± 0	77 ± 8	76	2.8 ± 0.1	40.6 ± 0.7	18.3 ± 3.1	23.4 ± 0.5	6.00	6.25	6.00	6.50
16	381 ± 1	11.5 ± 0.7	5.4 ± 0	0.1 ± 0.2	140 ± 8	73	2.91 ± 0.08	40.4 ± 0.8	16.3 ± 0.6	22.4 ± 0.4	6.50	6.00	6.50	6.00
17	384 ± 1	9.1 ± 0.3	5.67 ± 0.15	0 ± 0	90 ± 5	86	2.55 ± 0.11	40.2 ± 0.5	24.3 ± 5.5	18.5 ± 0.7	5.75	6.00	6.00	6.00
18	395 ± 1	6 ± 0.3	6.07 ± 0.06	0 ± 0	272 ± 8	76	3 ± 0.11	39.6 ± 0.3	12.7 ± 2.5	20.1 ± 0.8	6.50	6.50	6.00	6.25
19	398 ± 1	8.4 ± 0.6	5.33 ± 0.06	0 ± 0	287 ± 0	79	2.76 ± 0.07	39.8 ± 0.6	12.3 ± 3.5	21.3 ± 0.8	6.00	6.00	6.50	6.25
20	401 ± 5	12.3 ± 0.7	5.77 ± 0.06	0.2 ± 0.1	53 ± 6	64	3.64 ± 0.2	40.2 ± 0.2	41 ± 5	26 ± 1.4	6.00	5.50	7.00	6.00
21	408 ± 2	8.3 ± 0.5	5.83 ± 0.06	0.2 ± 0.4	95 ± 6	71	2.73 ± 0.06	40.2 ± 0.7	12.7 ± 2.9	19.5 ± 0.4	6.00	5.50	6.00	5.75
22	408 ± 4	13.2 ± 0.9	5.7 ± 0	0.6 ± 0.2	208 ± 12	65	2.9 ± 0.04	40.1 ± 0.1	31 ± 1.7	20.7 ± 0.6	5.75	6.00	6.00	6.00
23	411 ± 1	11.7 ± 0.4	5.37 ± 0.21	0.4 ± 0.2	150 ± 8	78	3.07 ± 0.1	39.9 ± 0.1	28 ± 4.6	23.3 ± 0.7	6.00	6.00	6.00	6.25
24	416 ± 3	8.9 ± 0.4	6.17 ± 0.06	0.1 ± 0.1	132 ± 5	65	3.3 ± 0.15	40.7 ± 0.9	15.3 ± 1.2	22.4 ± 0.5	6.00	6.00	6.00	6.00
25	422 ± 3	10.1 ± 0.4	5.8 ± 0.1	0.2 ± 0.3	160 ± 127	79	3.21 ± 0.01	40.3 ± 0.3	21.3 ± 2.1	22.9 ± 0.2	6.00	6.00	6.50	6.25
26	422 ± 3	10.1 ± 0.4	6.13 ± 0.06	0.1 ± 0.2	0 ± 6	66	3.47 ± 0.06	40 ± 0.1	21 ± 2	23.4 ± 0.5	6.00	6.00	6.00	6.00
27	428 ± 3	8.6 ± 0.5	4.97 ± 0.15	5.4 ± 1.3	113 ± 0	76	2.74 ± 0.05	40.7 ± 0.5	16.3 ± 1.2	22.9 ± 0.1	6.00	5.00	6.00	5.50
28	429 ± 2	13.7 ± 0.7	5.5 ± 0	0.5 ± 0.2	112 ± 0	76	2.54 ± 0.18	40.2 ± 0.4	30 ± 7.9	19.1 ± 1.5	6.00	6.00	6.50	6.00
29	429 ± 1	7.9 ± 0.1	5.3 ± 0	0 ± 0	70 ± 0	69	2.39 ± 0.26	40.6 ± 0.5	15.3 ± 2.9	18.8 ± 1.8	6.00	6.00	6.00	6.00
30	430 ± 2	8.4 ± 0.4	5.1 ± 0	0.1 ± 0	108 ± 6	80	2.89 ± 0.1	39.8 ± 0.1	20 ± 5.3	23.2 ± 0.8	6.25	6.25	6.00	6.00
31	432 ± 2	8.6 ± 0.4	5.9 ± 0.1	0 ± 0.1	38 ± 12	87	2.62 ± 0.07	40 ± 0.4	11.3 ± 0.6	18.4 ± 0.2	6.00	6.25	6.00	6.25
32	443 ± 1	8.3 ± 0.3	5.13 ± 0.06	0.2 ± 0.3	97 ± 3	93	2.95 ± 0.13	39.6 ± 1	13.3 ± 1.2	23.5 ± 1	6.00	6.00	6.00	6.00
33	445 ± 3	8.3 ± 0.4	5.03 ± 0.06	0 ± 0	93 ± 3	64	2.89 ± 0.05	40 ± 0.2	17.7 ± 2.5	23.7 ± 0.5	6.00	6.00	6.25	6.00
34	461 ± 7	9.1 ± 0.4	5.3 ± 0	0.1 ± 0.1	73 ± 6	84	2.7 ± 0.03	40.1 ± 0.2	12 ± 2	21.2 ± 0.3	6.00	6.25	6.00	6.25
35	461 ± 7	9.1 ± 0.4	5.17 ± 0.06	0.2 ± 0.3	278 ± 3	78	2.31 ± 0.18	40 ± 1.3	19 ± 5.6	18.3 ± 0.9	5.75	6.25	6.25	6.00
36	462 ± 2	8.3 ± 0.5	5.83 ± 0.06	0.1 ± 0.1	47 ± 8	85	2.79 ± 0.22	40 ± 0.2	28 ± 8.7	19.8 ± 1.5	6.00	6.00	5.75	5.75
37	476 ± 2	7.1 ± 0.4	5.13 ± 0.06	0.1 ± 0.1	88 ± 12	77	2.51 ± 0.06	40 ± 0.5	13.3 ± 1.5	20.2 ± 0.7	6.00	6.25	6.00	6.25
38	476 ± 3	12.1 ± 0.7	5.43 ± 0.06	0.2 ± 0.1	67 ± 10	67	2.9 ± 0.11	39.8 ± 0.2	38.7 ± 2.1	22 ± 1	6.00	6.00	6.00	6.00
39	490 ± 3	5.5 ± 0.3	5.6 ± 0.1	0.1 ± 0.1	0 ± 9	77	2.15 ± 0.07	39.7 ± 0.6	13.3 ± 2.1	15.7 ± 0.6	6.00	6.00	6.00	6.00
40	504 ± 2	5.3 ± 0.4	6.03 ± 0.06	0.1 ± 0.1	120 ± 13	81	2.66 ± 0.19	40.2 ± 0.4	16 ± 4.4	18.3 ± 1.1	7.00	7.00	7.00	6.50
41	519 ± 5	9.4 ± 0.3	5.83 ± 0.06	2.8 ± 0.1	180 ± 0	95	2.92 ± 0.14	40.1 ± 0.2	25.7 ± 5.7	20.7 ± 0.9	5.75	6.00	6.00	6.00
42	535 ± 7	8.8 ± 0.8	5.2 ± 0	20 ± 1.5	80 ± 0	71	2.4 ± 0.02	39.8 ± 0.1	21.3 ± 0.6	18.8 ± 0.1	5.75	6.00	5.75	6.00
43	549 ± 1	10.2 ± 0.6	5.13 ± 0.15	2.2 ± 3.5	38 ± 3	73	2.37 ± 0.15	40 ± 0.3	28.3 ± 8.7	19.1 ± 1.2	5.75	5.75	6.25	6.00

## Results and discussion

Defining espresso coffee and its quality is subject to discussion. Some link it to the concentration of solids found in the espresso brew^15^—the TDS. Others to the amount of water used per gram of coffee^16^—the brew ratio. Regardless of the perspective taken on the definition of what an espresso coffee is, the resulting low volume beverage should have a pleasant, distinct aroma, a complementary balanced profile between acid, bitter and sweetness as well as a pleasant tactile thickness, known as the body^15,16^.

As TDS will be heavily influenced by beverage volume, espresso brews prepared in this study were kept at a fixed beverage weight of 40 g. While brew weight was kept constant, the duration of extraction was allowed to vary and recorded to determine the average extraction flow rate.

In the following, linear and multivariant correlations of extraction variables will be discussed first. Second, quality and sensory aspects of the extraction process will be addressed.

### Characterizing the coffee grounds

Grinding coffee generates particles with a bimodal distribution^17^. One local maximum occurs at greater particle sizes^18^ and its location varies according to the adjustment (spacing) of the grinder burrs. For the sample in this study, this maximum is in the range 250–550 µm. The second local maximum occurs at smaller particle sizes, usually at around 50 µm. The particularity of this maximum is that its position seems to not be affected by the spacing of the grinding burrs. Median particle size rather than average was used in this study. The reason is that median particle size is less impacted by the amounts of fines, when such similar bimodal distributions are compared where the two peaks stay at the same position but only change in height.

**Figure 1:**
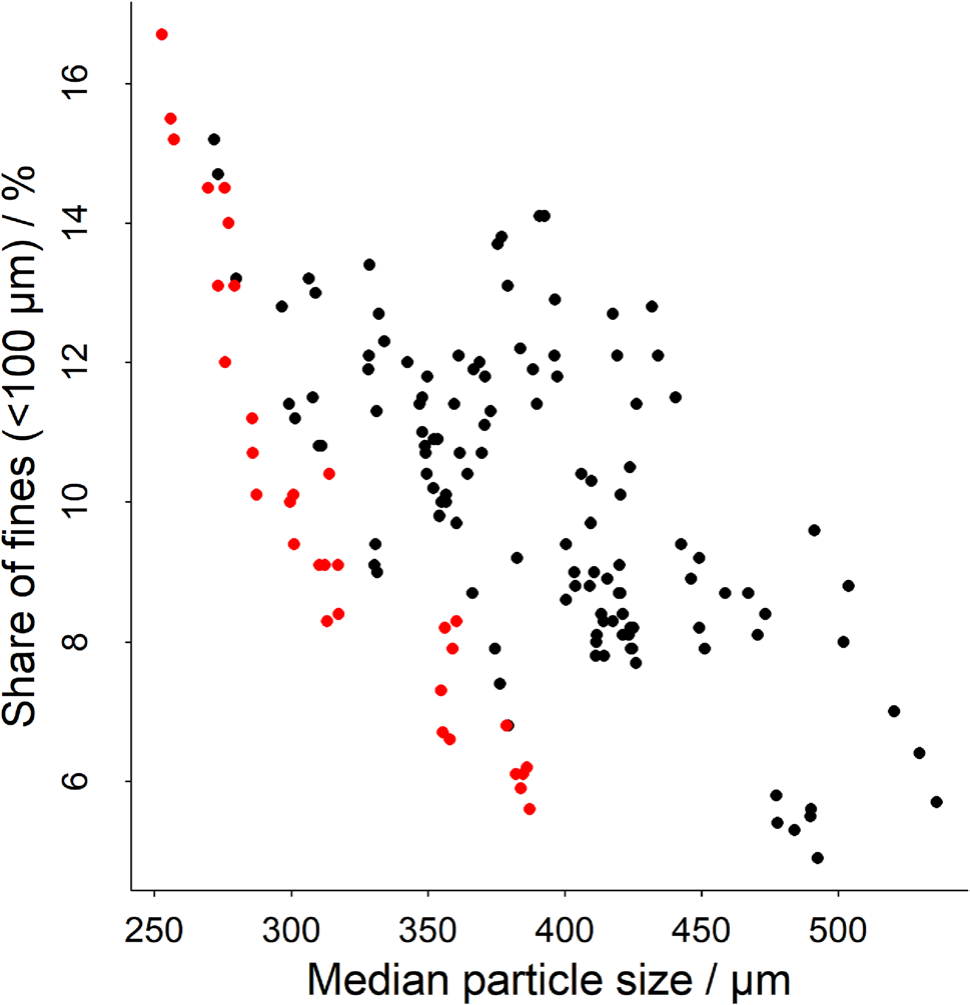
Volume median particle size of capsule coffee plotted against percent portion of fines. The samples highlighted in red show capsules from a single manufacturer.

In Fig. 1, the share of fine particles, Q_100µm_ is plotted as a function of the median particle size for the capsules measured in this study. The capsule coffee grinds show a wide spread in the amount (proportion) of fines, over the range from 6 to 16% of volume. Generally, there is a trend; as the coffee is ground more coarsely (larger median particle size), the shares of fines become smaller. In this study, a large variation in the share of fines for a given median particle size was found, which is most likely due to different capsule product lines. Coffees can have the same 10% share of fines while the median particle size can vary from 300 µm up to 550 µm. When only a single product line is considered (e.g. capsules from a single manufacturer; red dots, Fig. 1), a distinct grouping is observed. This result possibly reflects the grind technology used by this manufacturer, since the share of fines is clearly negatively associated with the median grind size, with a narrow spread of the data, compared to all the capsules tested in this study. For a specific median particle size, the capsules from this manufacturer systematically show the lowest proportion of fines. Furthermore, the proportion of fines increases in a linear fashion with decreasing median particle size.

### Influence of particle size distribution on extraction dynamics

**Figure 2:**
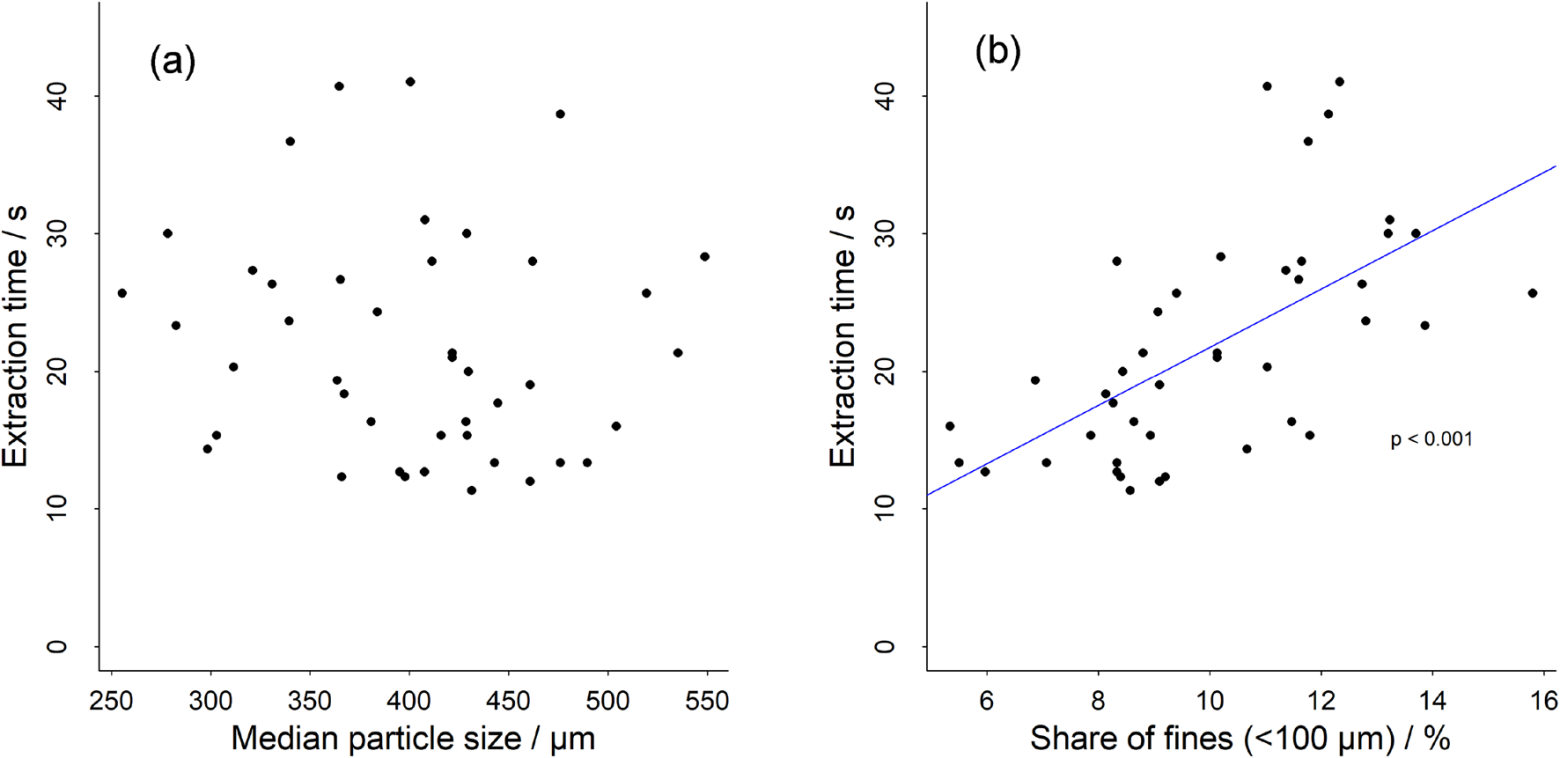
Correlations of median particle size (**a**) and share of fines (**b**) with extraction time.

Extraction times were used as an indirect reflection of the extraction dynamics. The extraction times were found to be highly variable between different types of capsules, covering a range between 10 and 41 s (Fig. 2), which corresponds to average flow rates in the 1–4 g/s range. When relating extraction times to particle size distribution, a highly significant positive association was found with Q_100µm_ (p < 0.001). Extraction time increased with increasing proportion of fines (Fig. 2b). More specifically, over the sample population an increase in the proportion of fines from 10 to 15% corresponded to an increase in the extraction time of 10 s. In contrast, the volume median particle size was not found to be correlated with the extraction time (Fig. 2a) as otherwise seems to be the case in the extraction with semi-automatic espresso machines^19,20^. Studies on the impact of the grind size for espresso extraction have mostly focused on the median or mean particle size^7,13,20^. The key variable that influences the hydrodynamics of espresso extraction is coffee bed permeability, which is a function of the particle size distribution of solids in the coffee bed and how the particles pack together to form the bed^19^. Hence, it is not only the median or mean particle size that plays a role in the permeability, but the overall particle size distribution also including the share of fine particles^19,21^.

Due to the large range of capsules and manufacturers included in this study, results are spread over a wide variety of Q_100µm_; X50 combinations (Fig. 1). Even though both parameters are related (Fig. 1), and that probably a range of different grinding technologies are used by different manufacturers, this study indicates that coffee bed permeability is impacted more by the share of fines in the capsule coffees than by the particle size itself. When grinding coffee on a single grinder type, both Q_100µm_ and X50 are changing simultaneously (for example Fig. 1, red dots), therefore extraction times will be related closely to the median particle size, as has been previously reported^19,20^. However, as Fig. 2 illustrates, the extraction time seems to be much more significantly impacted by the shares of fines than the median particle size.

**Figure 3:**
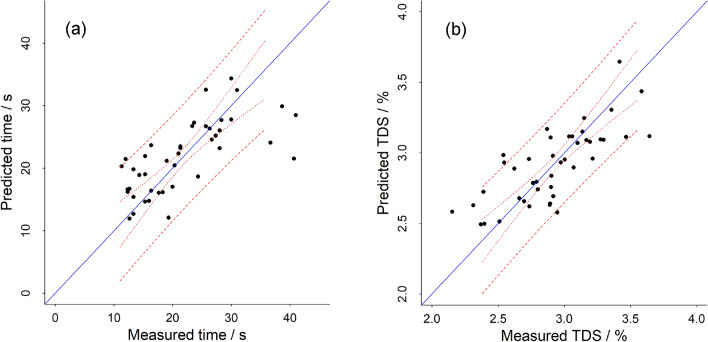
Multiple regression analysis models. (**a**) Prediction of extraction time by coffee weight, median particle size, share of fines, roast level and capsule pressure. (**b**) Prediction of TDS by coffee weight, median particle size, share of fines, roast level and capsule pressure. The dotted lines show 95% confidence interval and the dashed lines 95% prediction intervals.

In addition to searching for linear correlations, multiple regression analysis was applied to the data, in order to unveil multivariant correlations between capsule variables and extraction time (Fig. 3). Multiple regression analysis confirms what is observed with linear correlations. Extraction time as a function of coffee weight in the capsule, volume median particles size, roast level, share of fines and capsule pressure was modelled using multiple regression analysis. With such a model, 42% of the extraction time variance can be predicted, and the two parameters Q_100µm_ (p < 0.0001) and X50 (p < 0.01) have significant importance for the regression. The extraction time can be predicted based on the model (Fig. 3a), and the prediction works well for coffee extracting until 30 s extraction time, whereas for those that extracted longer than 35 s, the predicted values were smaller than the measured ones. The standardised model coefficient is double in value for Q_100µm_ than for X50 (Q_100µm_ 0.88, X50 0.45), and furthermore the expected coefficient for X50 would be negative, since smaller particle size could in theory only be associated with a longer extraction time. This result shows that there is some multicollinearity between share of fines and particle size, and confirms the higher importance of share of fines for controlling capsule extraction time, rather than particle size.

We suspected that higher capsule pressure would lead to longer extraction time, due to a larger amount of trapped carbon dioxide in the coffee bed, a residue from the degassing process^22^. No correlation of the capsule pressure with the extraction dynamics was observed.

### Influence of capsule coffee properties on the cup properties

Espresso quality may be viewed as a complex extraction puzzle where the proper ratio of desirable components must be extracted simultaneously, in a matter of seconds, in order to produce the most desirable product. One of the characteristics of espresso coffee is its viscous, thick appearance, which is caused by high concentration of coffee solubles (TDS)^15^. High TDS is usually a highly desired quality of the espresso brew and is related to the sensory perception of “body”. TDS is a non-specific analysis technique used routinely to gauge how much of the coffee was extracted into the cup, also called extraction yield.

**Figure 4:**
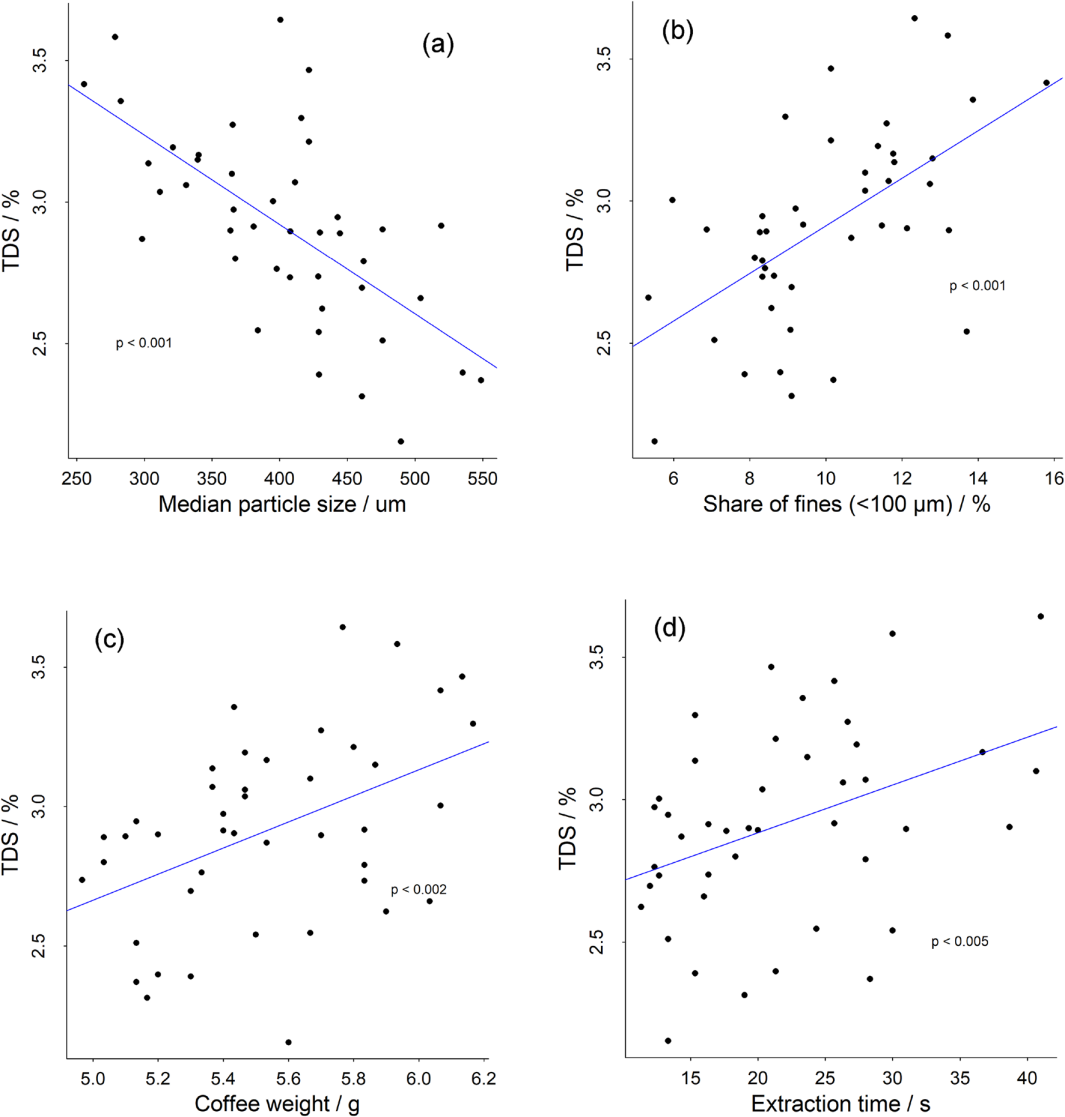
Correlations of median particle size (**a**), share of fines (**b**), weight (**c**), and time (**d**) with TDS.

Extraction time, coffee weight and proportion of fines were found to positively correlate with the TDS of the resulting brew (Fig. 4b-d). Negative correlation of the particle size with the TDS was found (Fig. 4a), which is expected and was reported before^23^. The extraction percentage—shows essentially the same relations with the variables mentioned above. This is because extraction percentage is calculated by multiplying TDS by brew weight and dividing by the coffee weight, and the brew weight was kept constant for all extractions in this study.

Unsurprisingly, the most direct relation and strongest correlation among those observed in this study is the weight of the coffee in the capsule with the TDS of the brew. Packing more coffee in the same extraction system will lead to a more concentrated brew when brewing to a fixed beverage weight. The linear relation observed follows the theoretical increase of TDS that should be observed when assuming the same extraction percentage from all capsule coffees (Fig. 4c). Indeed, over the whole range of capsules tested here, no relation of extraction percentage (in %) with the weight of coffee in the capsule was found (variable relation not shown).

Longer extraction times and hence slower flow rates are related to brews with higher TDS. The effect is surprisingly high, even though as reported by Melrose et al. the bed extraction efficiency theoretically does not increase significantly with longer extraction times^21^. The data from this study indicate a possible “saturation” effect at extraction times above 25 s (Fig. 4d). The extraction time and coffee weight show no significant correlation. The inverse correlation of TDS (and extraction percentage) with particle size is known and is explained by increased extraction efficiency from smaller particle size that enable the solubles to diffuse from the particles more quickly^21^. The complex correlation of variables observed here could also be impacted by the product specifications. Since consumers expect an espresso to be a thick and a strong brew, finer grind sizes with a higher share of fines are used to maximize the extraction percentage. Hence, long extraction times might be acceptable, and the pressure needed to extract the capsule pushed to the limit of the capsule machine by producers to achieve desired brew quality. We see some weak correlation (p < 0.05, data not shown) of TDS with roast level. This result may be attributed to the fact that the recipes for the capsules vary greatly, and darker roasted coffees could be preferred by consumer, as this leads to coffee with a stronger body. Hence, the correlation of roast level with TDS could not be attributed directly to better extractability of darker roasted coffee, since the grind properties play a more important role in the extraction behaviour.

Multiple regression analysis of TDS as a function of coffee weight, X50, Q_100µm_, roast level and capsule pressure (52.7% of TDS variance predicted) revealed that decreasing the particle size of the grounds, X50 and increasing the coffee weight, had the most prominent impact on TDS (standardized model coefficients -0.43 and 0.32, p < 0.01 and p < 0.01). The prediction of TDS based on the multiple regression model is shown in Fig. 3b. The authors are aware that the indicators of importance of variables (coefficient and p-value) in models are not completely robust, since the independent variables have clearly some multicollinearity. While building the multiple regression models it was found that the results are consistent, even when subsets of samples were modelled. This was the case when independent variables that were not important were excluded from the model, or other variables were included (for example, including time as an independent variable for the TDS model). Hence, it seems reasonable to conclude that the results from the multiple regression analysis revealed with good statistical confidence the importance of the variables predicting the TDS in the brew and the extraction time.

### Sensory analysis

As 43 different commercial capsule coffees were tested (Table 1) in this study, it was anticipated that flavour attributes would vary between products due to the potential use of different origins, varietals, processing methods, roast levels, grinds and blends. Nevertheless, body is particular as it represents a tactile sensation attributed to solids extracted from the coffee during brewing and becoming suspended in the beverage, making them quantifiable by measuring the TDS^24^.

Consequently, the sensory results have not shown correlations with technical variables apart from a suspected trend no significance of body correlated with TDS (p = 0.21). The Q-grading sensory evaluation was focused on the quality of the brew. The sensory results were mainly of values close to 6, since the coffee brews in this study did not reproduce the range of Q-grader results that could be expected for coffees prepared using a semi-automatic espresso machine and highest quality coffees. Some of the outliers in the sensory results could be explained by the parameters of the capsules. Two samples were found to have higher oxygen content in the capsule (samples 27 and 42) and scored lower in the sensory. Another two samples that were found to be higher than average in the sensory results (samples 19 and 40) could be explained to be of higher green coffee quality, since those samples were single origin Colombian and Ethiopia coffee. These sensory results and the lack correlation to technical parameters are not surprising since they show that most of the capsules have been optimised and produce average quality results. Only outliers in the sensory space were found as coffees of poorer production (oxygen in capsule) or coffees of above average green coffee quality.

**Figure 5:**
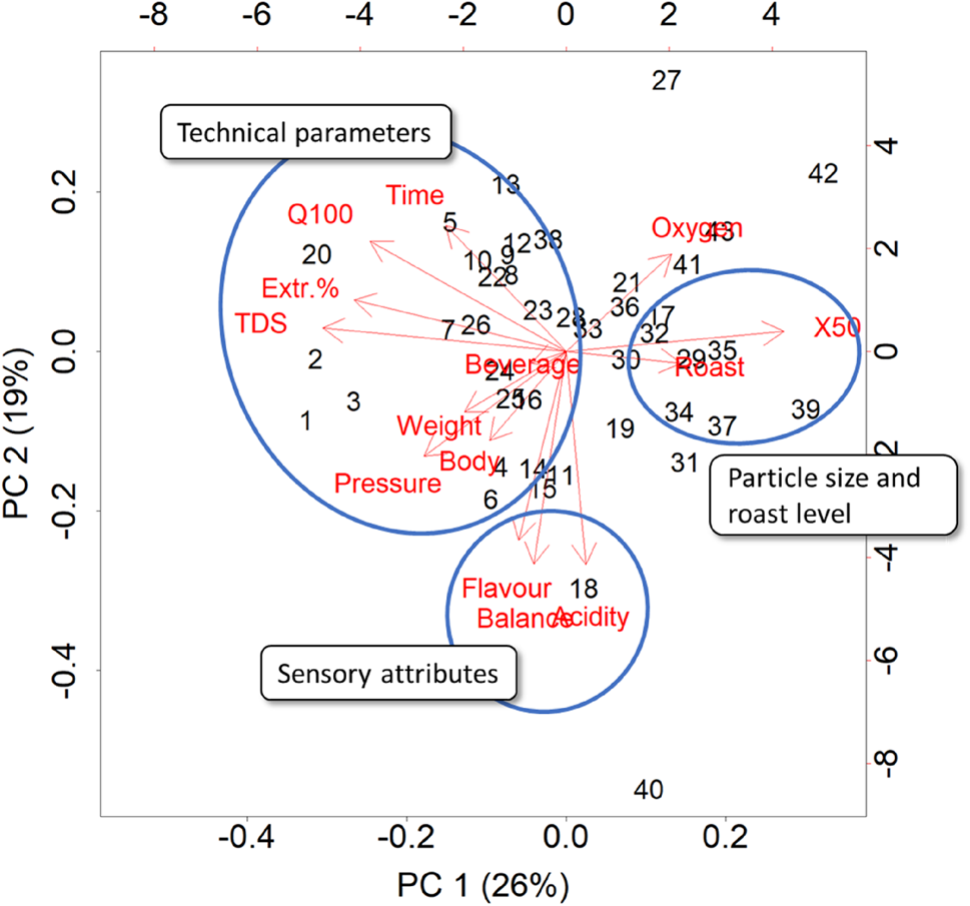
Principal component analysis of the capsule and brew data.

The orthogonality of sensory analysis results with capsule parameters is also shown by the principle component analysis (PCA), as displayed in Fig. 5. The differentiation of the capsule samples by PCA is caused by ground coffee properties in PC1 axis, together with the sensory descriptor body, which has the same direction as coffee weight along the PC1 axis. As expected, based on results discussed earlier, the loadings of particle size and roast level have the opposite value in PC1 direction to other loadings, since lower values of those have been found associated with higher values of other ground coffee variables and TDS. The remaining sensory attributes are oriented orthogonal to the technical variables, parallel to the PC2 axis, further confirming that the sensory quality properties of the brew within the capsule landscape of studied samples are not significantly impacted by the physical properties of the ground coffee and by variations in extraction variables. The samples mentioned in the previous paragraph (19, 27, 40 and 42) are also outliers on the PC2 axis and are likely the cause of the orthogonality of the sensory to technical parameters on the PCA plot.

## Conclusions

Despite the wide variety of different capsule coffee formulations used for production of capsules subjected to this study, we can draw some general conclusions with respect to the extraction of coffee from capsules and espresso brew properties as a function of ground coffee parameters. The capsule extraction dynamics are highly influenced by the particle size distribution of coffee grounds. Only mean or median particle size information is insufficient to understand capsule extraction, therefore in this study we propose the parameter “share of fines; Q_100µm_” as the second parameter for a simplified description of coffee grinds. The proportion of fines was highly correlated with increasing extraction time.

The best way to increase TDS is to use more coffee for extraction, or to grind the coffee more finely to increase extraction efficiency. Grinding finer to increase extraction comes with a technical problem; finer grind sizes tend to form more finer particles in the grounds, which cause a decreased permeability of the coffee bed. This study shows that while there are certain general rules that can be followed for capsule coffee product development, the fine-tuning of parameters for a successful capsule coffee extraction is a problem specific to capsule production line (e.g. different grinder technology produces different share of fines in the coffee grinds). The coffee sensory quality in the cup was not correlated to the technical variables of the grounds. It is suspected that because most of the samples used in this study are optimised for the extraction, the cup quality can be only further improved by using higher quality green coffee. To better understand in what range the optimisation of technical parameters and green coffee quality affect brew quality, future studies with capsules produced using a controlled range of conditions need to be conducted.

## References

[1] Bartel C, Mesias M, Morales FJ (2015). Investigation on the extractability of melanoidins in portioned espresso coffee. Food Res. Int..

[2] Parenti A (2014). Comparison of espresso coffee brewing techniques. J. Food Eng..

[3] Petisca C, Perez-Palacios T, Pinho O, Ferreira I (2014). Optimization and application of a HS-SPME-GC-MS methodology for quantification of furanic compounds in espresso coffee. Food Anal. Methods.

[4] Gloess AN (2013). Comparison of nine common coffee extraction methods: instrumental and sensory analysis. Eur. Food Res. Technol..

[5] Candeias SX, Gallardo E, Matos AC (2009). caffeine content of retail market coffee in Portugal. Food Anal. Methods.

[6] Lopes GR (2016). Carbohydrate content, dietary fibre and melanoidins: composition of espresso from single-dose coffee capsules. Food Res. Int..

[7] Wang XJ, William J, Fu YC, Lim LT (2016). Effects of capsule parameters on coffee extraction in single-serve brewer. Food Res. Int..

[8] Monakhova YB (2015). Rapid approach to identify the presence of Arabica and Robusta species in coffee using H-1 NMR spectroscopy. Food Chem..

[9] Rahn A, Yeretzian C (2019). Impact of consumer behavior on furan and furan-derivative exposure during coffee consumption. A comparison between brewing methods and drinking preferences. Food Chem..

[10] Angelino D, Tassotti M, Brighenti F, Del Rio D, Mena P (2018). Niacin, alkaloids and (poly)phenolic compounds in the most widespread Italian capsule-brewed coffees. Sci. Rep..

[11] Gloss AN, Schonbachler B, Rast M, Deuber L, Yeretzian C (2014). Freshness indices of roasted coffee: monitoring the loss of freshness for single serve capsules and roasted whole beans in different packaging. Chimia.

[12] Cozzolino CA, Pozzoli S, La Vecchia S, Piergiovanni L, Farris S (2015). An alternative approach to control the oxygen permeation across single-dose coffee capsules. Food Pack. Shelf Life.

[13] Severini C (2016). How the variance of some extraction variables may affect the quality of espresso coffees served in coffee shops. J. Sci. Food Agric..

[14] Sanchez-Lopez JA, Zimmermann R, Yeretzian C (2014). Insight into the time-resolved extraction of aroma compounds during espresso coffee preparation: online monitoring by PTR-ToF-MS. Anal. Chem..

[15] Petracco, M. The Cup. In *Espresso Coffee: The Chemistry of Quality* (eds. Illy A. & Viani R.) (Academic Press, Cambridge, 1995) p. 187.

[16] Mestdagh, F., Glabasnia, A. & Giuliano, P. In *The Craft and Science of Coffee* (ed Britta Folmer) 355–380 (Academic Press, 2017).

[17] Macedo, P. H. Percolation. In *Espresso Coffee: The Science of Quality* (eds. Illy A. & Viani R.) (Elsevier, 2005) p. 277.

[18] von Blittersdorff, M. & Klatt, C. In *The Craft and Science of Coffee* (ed Britta Folmer) 311–328 (Academic Press, 2017).

[19] Corrochano BR, Melrose JR, Bentley AC, Fryer PJ, Bakalis S (2015). A new methodology to estimate the steady-state permeability of roast and ground coffee in packed beds. J. Food Eng..

[20] Severini C, Ricci I, Marone M, Derossi A, De Pilli T (2015). Changes in the aromatic profile of espresso coffee as a function of the grinding grade and extraction time: a study by the electronic nose system. J. Agric. Food Chem..

[21] Melrose J, Roman-Corrochano B, Montoya-Guerra M, Bakalis S (2018). Toward a new brewing control chart for the 21st century. J. Agric. Food Chem..

[22] Smrke S (2018). Time-resolved gravimetric method to assess degassing of roasted coffee. J. Agric. Food Chem..

[23] Andueza S, De Pena MP, Cid C (2003). Chemical and sensorial characteristics of espresso coffee as affected by grinding and torrefacto roast. J. Agric. Food Chem..

[24] Andueza S, Vila MA, de Pena MP, Cid C (2007). Influence of coffee/water ratio on the final quality of espresso coffee. J. Sci. Food Agric..

